# Impact of a new combined preoperative cleft assessment on dental implant success in patients with cleft and palate: a retrospective study

**DOI:** 10.1186/s12903-022-02040-5

**Published:** 2022-03-15

**Authors:** Charles Savoldelli, Sonanda Bailleux, Emmanuel Chamorey, Clair Vandersteen, Barbara Lerhe, Franck Afota

**Affiliations:** 1 Oral and Maxillo-Facial Surgery Department, Head and Neck Institute, University Hospital of Nice, 30 Avenue Valombrose, 06100 Nice, France; 2 Paediatric Maxillofacial Surgery and ENT Department, Lenval Hospital, Nice, France; 3 Clinical Research, Innovation and Statistics Department, Centre Antoine Lacassagne, Nice, France

**Keywords:** Alveolar cleft score, Cleft lip, Cleft palate, Dental implant, Interdental alveolar bone height, Patient compliance, Patient satisfaction

## Abstract

**Background:**

Bone height assessment alone is frequently used to guide rehabilitation choice, without consideration for soft tissues or adjacent teeth. This study aimed to evaluate the impact of different preoperative cleft assessments on implant success and patient satisfaction.

**Methods:**

The study involved a retrospective assessment of records from 40 patients with cleft lip and palate (CLP). The alveolar cleft score (ACS; clinical criteria), interdental alveolar bone height (IABH) score (radiological criteria), patient compliance score (dental hygiene, medical visit observance, and smoking), and a novel combined score (IABH-ACS-Compliance) were assessed from patient records. Patients who required prosthetic tooth rehabilitation in the cleft dental arch space were included. Twenty-six patients (Group 1) were treated with dental implants, and 14 patients (Group 2) selected another prosthetic option (fixed prosthodontics, removal prosthesis), orthodontic space closure, or no rehabilitation. The main outcomes measured were relative implant success (no implant loss involving marginal bone loss ≤ 1.9 mm) for patients treated with dental implant therapy (Group 1) and patient satisfaction for all patients (Groups 1 and 2).

**Results:**

Forty dental implants were placed in the patients in Group 1. Four implants in four patients (Group 1 relative failure, RF) were lost (implant survival rate of 90%) after 36 (± 12.4) months of follow-up. Twenty-two patients who received implants belonged to the relative implant success group (Group 1 RS). The average “IABH-ACS-Compliance” scores were significantly different (*p* < 0.05): 16.90 ± 2.35 and 12.75 ± 0.43 for the Group 1 RS and RF groups, respectively.

**Conclusions:**

Preoperative cleft parameters have an impact on relative implant success and patient satisfaction. The new cleft assessment combined-score (“IABH-ACS-Compliance”) allows an accurate selection of cleft cases eligible for dental implants, thereby improving postoperative outcomes.

## Background

Cleft lip and palate (CLP) are the most common congenital craniofacial abnormalities, affecting approximately 1.5–1.7 of every 1000 infants born, with ethnic and geographic variation [1]. During growth, treatment of CLP requires a multidisciplinary approach because of its multiple functional consequences, including feeding, facial growth, audiologic, and speech complications. In addition to functional cleft effects, important tooth disorders have been reported in patients with CLP, including anomalies in the number (agenesis), shape (microdontia or taurodontism), and position of teeth [2–4]. These disorders primarily involve the lateral maxillary incisors. Indeed, tooth agenesis is frequently described in combination with cleft lip and/or palate (CL/P). The prevalence of tooth agenesis in patients with complete unilateral CLP ranges from 48.8 to 75.9% and 27.2 to 48.8% inside and outside, respectively, the cleft region [5–7]. Disturbances during embryogenesis, as well as possible iatrogenic interferences during surgical interventions in the initial phase of tooth formation are generally responsible.

At the end of growth, rehabilitation of this edentulous space is an important phase of treatment that involves re-establishing esthetics, phonetics, and function. Dental prosthetic rehabilitation options include conventional prostheses (i.e., removable partial dentures and fixed partial dentures), orthodontic space closure, or implant-supported prostheses. These four options are largely discussed in the literature and have their advantages and disadvantages [8, 9].

Removable prostheses are a historic solution used today as a provisional option. However, this option permits an immobilization system that awaits definitive rehabilitation and prevents adjacent tooth movements.

Fixed prosthodontics (crown, bridge, veneers, or cantilever) can also be used for dental rehabilitation [10]. This prosthetic option incurs a great number of failures because of the high mobility of the teeth adjacent to the maxillary fragments [11]. Furthermore, this option does not allow functional loading of the bone graft, thereby facilitating progressive graft resorption. In addition, the use of a fixed prosthesis often results in the mutilation of adjacent healthy teeth [12, 13].

Orthodontic space closure permits a simplified process and earlier continuity of the dental arch. Nevertheless, space closure results in occlusal instability [14] and arch perimeter reduction, which aggravates the tendency for brachymaxilly.

Since Verdi et al. [15] reported the first case, dental implants have represented a reliable treatment option with great esthetic results. Several studies have discussed implant treatment for the cleft area and noted that this solution shows high success rates in the long term, [11, 16–20] similar to findings in patients without clefts [21]. Dental implants allow the loading of bone grafts. Nevertheless, dental implants for CLP rehabilitation require an experienced interdisciplinary team with respect to the conditions of implant fixation, which include adequate bone quantity and quality for implant placement, a favorable oral environment, and good patient compliance.

The interdental alveolar bone height (IABH) score is generally used to evaluate the bone level before implant placement in patients with CLP [17]. This score indicates whether a patient is eligible for an implant based solely on the bone height conditions. The alveolar cleft score (ACS), a clinical index introduced by Molé and Simon [22], allows a wider oral cleft evaluation. The ACS describes seven tissue indicators that are considered important for the management of alveolar sequelae and assigns them individual scores (range, 0–2). The seven tissue indicators are the width of the prosthetic space, nature of the lateral incisor, state of the adjacent teeth (i.e., corono-radicular state), quality of the periodontium, depth of the cleft epithelial invagination, quantity of the keratinized gingiva, and dimensions of the alveolar bone. The preoperative final score, obtained by adding the individual scores, can assume a low, high, or maximum value (range, 1–14). In contrast to the ACS, which provides a clinical cleft overview, the IABH score focuses only on bone height evaluation. According to our clinical experience, patient compliance can have a positive or negative impact on a patient’s therapeutic orientation, especially for patients with CLP who would have undergone multiple surgeries since birth. Furthermore, smoking can be a source of bone graft resorption and implant failure [23], which influence the final rehabilitation choice. The present retrospective study aimed to examine the effect of different preoperative assessments (IABH score, ACS, and compliance score) on dental implant success and final satisfaction in patients with CLP. The final goal was to develop a new scoring system that could identify the best method for dental rehabilitation of the residual edentulous space in patients with clefts.

## Methods

Forty patients with alveolar clefts were included in this retrospective study. All patients had a residual edentulous cleft space due to lateral incisive agenesis or avulsion of a microdontic tooth.

The strategy method for the treatment of patient with alveolar cleft involved multi-disciplinary decision in growth stages. The means of reconstruction of the anterior alveolar defect was secondary bone grafting at the age of 8–11 years, while the mixed dentition was present.

Alveolar bone reconstruction was performed with a bone graft, using a gingivoperiosteoplasty technique, during the mixed dentition stage and before permanent canine eruption. Secondary bone grafts were harvested from the anterior iliac crest, using particles of cancellous bone and marrow (24 patients), or from the parietal bone, using bone particles of cortical and cancellous bone (3 patients). Tertiary bone grafting was performed in fully grown patients starting at the age of 17 if needed according to the previous technique.

The restoration of missing teeth in cleft patients with orthodontic gap closure, conventional prosthetics (fixed, removable, or overlay), or dental implants was decided and performed in this stage of fully growth patient.

A single specialized examiner collected all information from patient medical records, pictures, and radiographs. Regardless of the type of rehabilitation, except for one patient who was treated with Disk-implant® (Victory, France) and was excluded, all patients with clefts and dental rehabilitation of the edentulous space treated from 2016 to 2019 were included in this study. Preoperative data were registered for all patients who underwent CLP management. The following scores were used in this study:

1. Alveolar left score (range, 1–14) derived from Molé et Simon’s score [22]
2. Interdental-alveolar bone height score (range, 0–4) determined according to Takahashi et al. [17] and adapted from Abyholm and Bergland [24, 25].
3. Compliance score (range, 0–3).

**Fig. 1:**
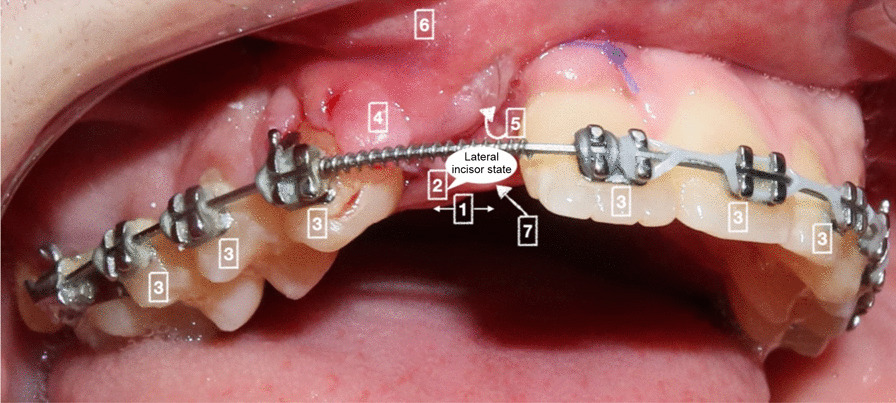
Alveolar cleft score which evaluates 7 parameters. A score range of 0 (unfavorable), 1 (possible with local management), or 2 (favorable) is assigned to each parameter. The parameters are the (1) prosthetic space (width), (2) lateral incisor (presence, shape, or anomaly), (3) bordering teeth (with root state), (4) bordering periodontium, (5) epithelial invagination (depth), and (6) buccal surface (regularity) and palatal mucosa (inflammation, fistulae)

The ACS (Fig. 1) is a clinical cleft tissue assessment that evaluates seven parameters: the prosthetic space (width), lateral incisor (presence, shape, or anomaly), bordering teeth (with root state), bordering periodontium, epithelial invagination (depth), buccal surface (regularity), and palatal mucosa (inflammation, fistulae). A score of 0 (unfavorable), 1 (possible with local management), or 2 (favorable) is assigned to each parameter.

**Fig. 2:**
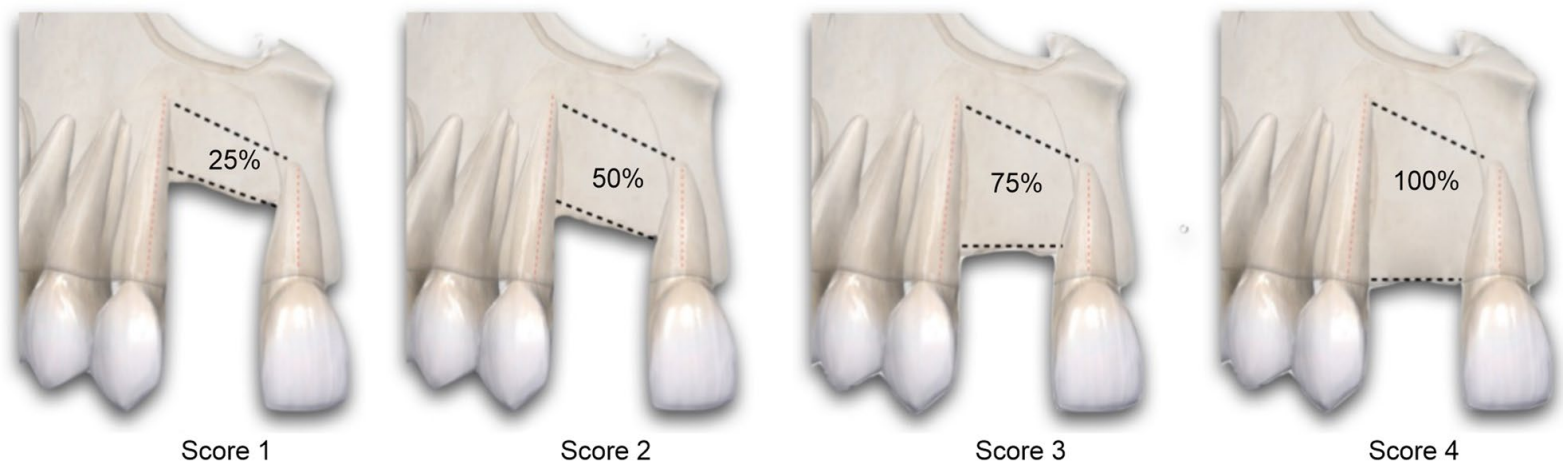
Interdental alveolar bone height assessment

The IABH score (Fig. 2) was determined in relation to the interdental bone height and assessed on a 4-point scale: 1 (75–100% bone loss), 2 (50–75% bone loss), 3 (25–50% bone loss), and 4 (0–25% bone loss). If the IABH score was estimated to be 3 or 4, cone-beam computer tomography is realized, and the implant could be placed according to the 3D bone volume assessment. If the score was < 2, the patient received a secondary bone graft. The compliance score (range, 0–3) was assigned by two professional examiners, summing dental hygiene (0, good; 1, bad), medical visit observance (0, good; 1, bad), and smoking (0, nonsmoker; 1, smoker). Age and sex were recorded. By adding the ACS, IABH score, and compliance score data, a new combined score named “IABH-ACS-Compliance” (range, 0–21), was suggested to synthesize the oral cleft environment at the time of prosthetic orientation selection.

Subsequently, the edentulous maxillary cleft space was rehabilitated according to a multidisciplinary discussion on mesiodistal space, bone and soft tissue evaluation, occlusion, and patient motivation. The following prosthetic treatments were proposed for the edentulous space: dental implants, fixed prosthodontics, removable dentures, and orthodontic space closure. Patient series was divided in group of patients with dental implant (Group 1) and group of patients with other rehabilitation of the missing tooth space (Group 2) and results of IABH, ACS and compliance were analyzed for each group. After 3-years follow-up, patient satisfaction was assessed in group 1 and group 2, the implant failure and the marginal bone lost (MBL) were assessed in group 1. To proceed with implant surgery, patients underwent cone-beam computed tomography (CBCT) scanning and retro-alveolar radiography examination. A parallel radiography technique was used: the film was placed parallel to the long axis of the neighboring tooth, and the central X-ray beam was directed perpendicular to the long axis of the tooth.

**Fig. 3:**
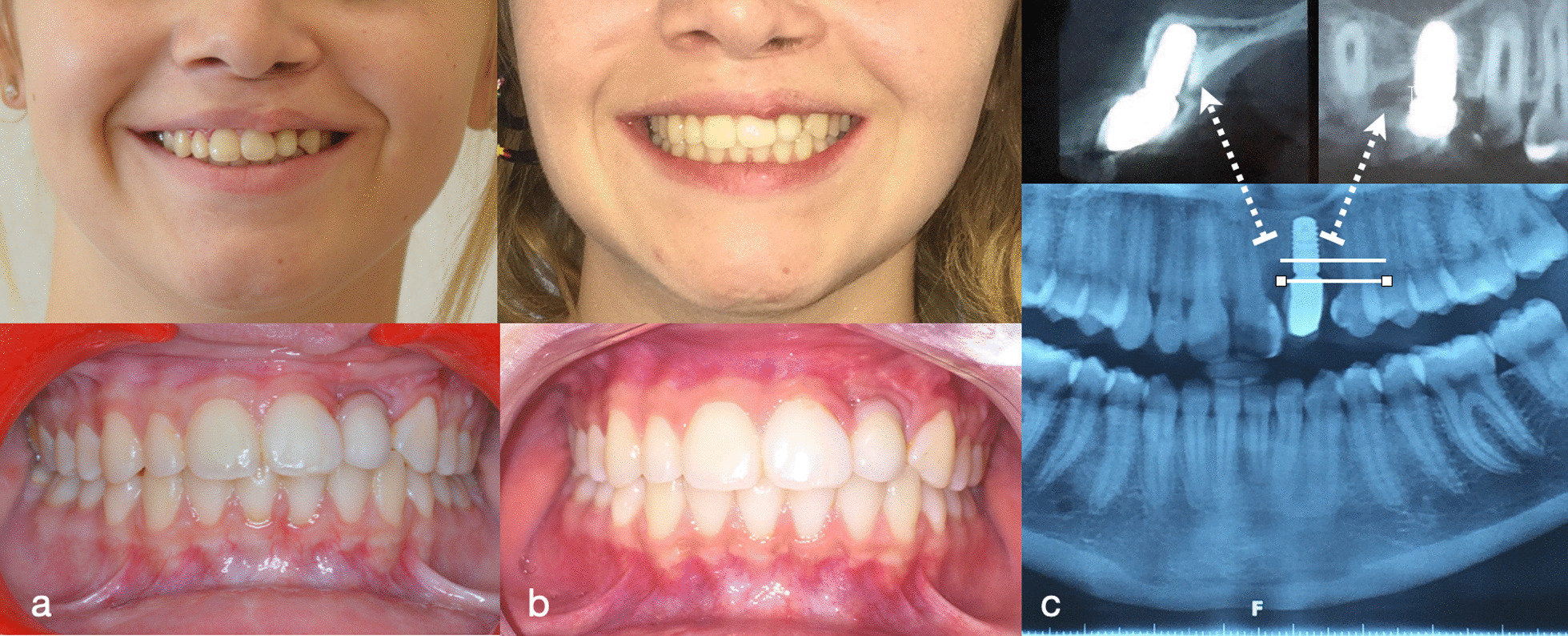
Patient clinical and radiologic follow-up. **a** 12 months clinical follow-up, **b** 3 years clinical follow-up, and **c** 3 years radiologic follow-up with MBL assessment. MBL, marginal bone loss

Using the CBCT reconstructed images, we selected implants with the proper diameter and length, considering the available bone (width and length). The interval between implant placement and last grafting (horizontal, vertical, or both) was also recorded. Commercially available pure titanium screw implants with a smooth surface and neck were placed: 15 Zimmer Dental Tapered Screw-Vent® (Zimmer Dental, Carlsbad, USA) and 11 Straumann Bone Level® (Institute Straumann, Waldenburg, Switzerland). The number of implants placed in each patient ranged widely depending on the type of cleft and space between teeth adjacent to the cleft area (including cases of infant premaxillary necrosis). Implant placement was performed in accordance with the recommended submerged surgical protocol. The implant shoulder level was equal to that of the bone crest. Preoperative broad-spectrum antibiotics (amoxicillin 2 g) were administered and continued for one week. All implants were allowed to integrate for four months before implant exposure and subsequent prosthodontic procedures. Soft tissue management around the implants was performed in 24 patients at the second surgery or later. This was done to improve peri-implant conditions. The follow-up period was 36 months after implant surgery or other prosthetic rehabilitations. After three years of follow-up, three data records were complete: implant survival, marginal bone loss (MBL), and patient satisfaction (Fig. 3).

Implant survival was recorded and used to calculate the implant survival rate. Marginal bone loss was evaluated using calibrated CBCT and was defined by the distance, in millimeters, from the implant shoulder to the alveolar crest [26]. The criteria proposed by Albrektsson et al. [27] served as a baseline for evaluating implant success, stipulating that vertical bone loss for osseointegrated implants should be 1.5 mm for the first year, and < 0.2 mm annually thereafter. Consequently, in this study, we considered an MBL of 1.9 mm as the threshold value (1.5 mm for the first year and 0.4 mm for the second and third years).

The patients with implants (group 1) in this study were divided into two groups: “Relative Success” (RS) group (no implants were lost and the MBL was ≤ 1.9 mm after 3 years) and “Relative Failure” (RF) group (one or several implants were lost or the MBL was > 1.9 mm after 3 years).

Final patient satisfaction was measured using the implant crown esthetic index (range, 0–5) [28]. The implant crown esthetic index was well adapted for patients with CLP compared with the pink esthetic score, which was judged too strict for CLP patients. Satisfaction was also evaluated in group 2 (other rehabilitation). The evaluation was performed by the patients themselves and by professional examiners (two maxillofacial surgeons and two dentists).

### Ethical approval and consent

All data were retrospectively collected and analyzed from routine cleft procedures. Informed consent for the use of anonymized data from medical records was obtained from the patients participating in the study and their legal representatives. The Institutional Review Board of Head and Neck University Institute (IRB No. 2017–05) approved the study protocol. In addition, our monocentric research on retrospective data meets the requirements of the MR003 (Reference Method 003), number 2202706, of the Commission Nationale de l’Informatique et des Libertés [National Commission for Data Protection].

### Statistical analysis

Qualitative data are presented as absolute and relative frequencies and were compared according to the χ^2^ test or Fisher’s exact test, as necessary. Quantitative data are presented as means and standard deviations, or medians and ranges, and were compared using the Student’s t-test or the Wilcoxon test. The normality of the distribution was tested using the Kolmogorov–Smirnov test. A 3-point difference between mean scores was considered clinically relevant. Considering this hypothesis, the inclusion of 40 patients yielded 90% power for the study. All statistical analyses were two-sided, and a p-value < 0.05 was considered statistically significant. All calculations were performed using R 3.2.2 software.

## Results

Of the 40 (22 male and 18 female) patients included in this study, 14 had unilateral cleft lip and alveolus (CLA) (10 left CLA and 4 right CLA), 19 had unilateral cleft lip, alveolus, and palate (CLAP) (14 left CLP and 5 right CLAP), and 7 had bilateral CLAP. At the time of prosthetic orientation selection, the average age of the patients was 20.72 (range, 16–28).

A total of 26 patients were rehabilitated by dental implants, whereas 14 were rehabilitated by other systems (fixed prosthesis, 5; removal dentures, 2; orthodontic closure, 5; and 2 patients did not receive any prosthetic rehabilitation). A total of 40 implants were placed into the cleft site in 26 patients (Table 1).

**Table 1:** Preoperative data of the 40 patients

Patient	Gender	Age	Type of Cleft	Rehabilitation	ACS score	IABH score	Compliance	Implant company	Implant size	Sum ACS-IABH-compliance
1	M	17	CLA Unilat Left	Implant	13	4	3	Zimmer TSV	Diameter: 3.7 mm Length: 10 mm	20
2	M	16	CLP Unilat Left	Implant	10	3	3	Straumann BL	Diameter: 3.3 mm Length: 10 mm	16
3	F	17	CLA Unilat Left	Implant	12	3	3	Straumann BL	Diameter: 3.3 mm Length: 10 mm	18
4	M	16	CLP Unilat Left	Implant	8	2	2	Straumann BL	Diameter: 3.3 mm Length: 12 mm	12
5	M	20	CLP Bilat	Implant	8	2	2	Zimmer TSV	Diameter: 3.7 mm Length: 10 mm	12
6	M	19	CLP Bilat	4 implants	9	2	3	Straumann BL	Diameter: 3.3 mm Length: 8 mm	14
7	F	24	CLP Unilat Left	2 implants	10	2	3	Straumann BL	Diameter: 3.3 mm Length: 8 mm	15
8	M	28	CLP Unilat Left	Implant	9	2	2	Zimmer TSV	Diameter: 3.7 mm Length: 10 mm	13
9	F	25	CLA Unilat Left	Veneers and cantilever	8	3	0	–	–	11
10	M	18	CLP Unilat Left	Orthodontic closure	7	2	0	–	–	9
11	F	25	CLA Unilat Left	Bridge	6	0	1	–	–	7
12	F	18	CLP Unilat Left	No rehabilitation	4	0	0	–	–	4
13	F	25	CLP Bilat	Removable denture	2	0	0	–	–	2
14	M	19	CLP Unilat Right	Implant	13	3	3	Zimmer TSV	Diameter: 3.7 mm Length: 10 mm	19
15	M	24	CLP Bilat	4 implants	10	3	3	Straumann BL	Diameter: 3.3 mm Length: 10 mm	16
16	M	22	CLA Unilat Right	Implant	12	3	1	Straumann BL	Diameter: 3.3 mm Length: 8 mm	16
17	F	17	CLA Unilat Left	Implant	9	2	2	Straumann BL	Diameter: 3.3 mm Length: 8 mm	13
18	M	20	CLA Unilat Right	Implant	13	3	3	Zimmer TSV	Diameter: 3.7 mm Length: 10 mm	19
19	M	26	CLP Unilat Right	Orthodontic closure	6	2	1	–	–	9
20	M	21	CLP Unilat Left	Bridge	2	1	1	–	–	4
21	F	25	CLA Unilat Left	Bridge	6	0	1	–	–	7
22	M	18	CLP Unilat Left	No rehabilitation	4	0	0	–	–	4
23	F	25	CLP Bilat	Removable denture	2	1	0	–	–	3
24	M	19	CLP Unilat Right	Implant	13	4	3	Zimmer TSV	Diameter: 3.7 mm Length: 8 mm	20
25	F	24	CLP Bilat	4 implants	11	3	3	Straumann BL	Diameter: 3.3 mm Length: 10 mm	17
26	F	21	CLA Unilat Right	Implant	8	3	1	Straumann BL	Diameter: 3.3 mm Length: 10 mm	12
27	F	20	CLA Unilat Right	Implant	13	3	3	Zimmer TSV	Diameter: 3.7 mm Length: 8 mm	19
28	F	23	CLP Unilat Right	Orthodontic closure	5	2	1	–	–	8
29	M	21	CLP Unilat Left	Bridge	3	1	1	–	–	5
30	M	17	CLA Unilat Left	Implant	13	4	3	Zimmer TSV	Diameter: 3.7 mm Length: 10 mm	20
31	F	18	CLP Unilat Left	Implant	12	3	3	Zimmer TSV	Diameter: 3.7 mm Length: 8 mm	18
32	M	18	CLA Unilat Left	Implant	12	3	3	Zimmer TSV	Diameter: 3.7 mm Length: 10 mm	18
33	M	16	CLP Unilat Left	Implant	11	4	3	Zimmer TSV	Diameter: 3.7 mm Length: 10 mm	18
34	M	20	CLP Bilat	4 implants	11	3	2	Zimmer TSV	Diameter: 3.7 mm Length: 11.5 mm	16
35	F	19	CLA Unilat Left	Implant	9	2	1	Zimmer TSV	Diameter: 3.7 mm Length: 8 mm	13
36	M	26	CLP Unilat left	2 implants	10	2	3	Straumann BL	Diameter: 3.3 mm Length: 8 mm	15
37	F	20	CLP Unilat Left	Implant	9	3	3	Zimmer TSV	Diameter: 3.7 mm Length: 11.5 mm	15
38	M	25	CLA Unilat Left	Orthodontic closure	8	3	0	–	–	11
39	F	18	CLP Unilat Left	Orthodontic closure	7	2	0	–	–	9
40	F	19	CLP Unilat Right	Implant	12	4	3	Zimmer TSV	Diameter: 3.7 mm Length: 11.5 mm	19

ACS, alveolar cleft score; IABH, interdental alveolar bone height; Compliance, dental hygiene, medical visit observance, and smoking; CLA, cleft lip and alveolus; CLP, cleft lip and palate, Unilat, unilateral; Bilat, bilateral

The average ACS for all patients was 8.75 ± 3.32 (range, 2–13). The mean IABH score was 2.3 ± 1.16 (range, 0–4). Mean compliance was rated at 1.85 ± 1.19 (range, 0–3). The average combined score “ACS-IABH-Compliance” was 12.90 ± 5.33 for all patients (range, 2–20).

There was a significant difference (*p* < 0.05) between the average “ACS-IABH-Compliance” scores for patients rehabilitated by dental implants (16.26 ± 2.63) and other types of rehabilitation, including 12 patients without any rehabilitation (6.64 ± 2.86; Table 2).

**Table 2:** Mean (SD) scores in implant patients (Group 1) and other rehabilitation patients (Group 2)

	Groupe 1 n = 26	Group 2 n = 14	Total N = 40	Test	*p* value
ACS	10.77 (1.73)	5 (2.1)	8.75 (3.32)	Student *t*-test	0.178
IABH	2.88 (0.68)	1.21 (1.08)	2.3 (1.16)	Student *t*-test	0.124
Compliance	2.6 (0.62)	0.42 (0.49)	1.85 (1.19)	Wilcoxon	0.03
Combined score (IABH-ACS-Compliance)	16.26 (2.63)	6.64 (2.8)	12.9 (5.33)	Student *t*-test	0.009

ACS, alveolar cleft score; IABH, interdental alveolar bone height; Compliance, dental hygiene, medical visit observance, and smoking

Twenty-two and four implanted patients belonged to the RS and RF groups, respectively (Table 3). There was a significant difference between the average “ACS-IABH-Compliance” scores for the RS and RF groups (16.90 ± 2.35 and 12.75 ± 0.43, respectively; *p* < 0.05; Table 2).

**Table 3:** Mean (SD) scores in the relative success and failure groups of Group 1

Score	Group 1 RS n = 22 Mean (SD)	Group 1 RF n = 4 Mean (SD)	Test	*p* value
ACS	11.13 (1.63)	8.75 (0.43)	Student *t*-test	0.155
IABH	3.04 (0.63)	2 (0)	Student *t*-test	0.297
Compliance	2.72 (0.61)	2 (0)	Wilcoxon	0.007
Combined score (IABH-ACS-Compliance)	16.09 (2.35)	12.75 (1.7)	Student *t*-test	0.006

ACS, alveolar cleft score; IABH, interdental alveolar bone height; Compliance, dental hygiene, medical visit observance, and smoking; RS, relative success; RF, relative failure; SD, standard deviation

Three years after dental rehabilitation, 4 implants (of the 40 that were placed) were lost (implant survival rate of 90%) and the average MBL was 0.81 mm ± 0.87 (range, 0.1–3.4; Table 4).

**Table 4:** Patient satisfaction, implants lost, and marginal bone loss measured after 3 years of follow-up

Patient	Satisfaction	Implants lost	MBL (mm)	Group (RS/RF)
1	5	0	0.25	RS
2	4	0	1.1	RS
3	3	0	0.1	RS
4	3	1	X	RF
5	4	0	3.4	RS
6	5	0	0.3	RS
7	4	0	0.85	RS
8	4	1	X	RF
9	5	X	X	X
10	3	X	X	X
11	4	X	X	X
12	2	X	X	X
13	2	X	X	X
14	5	0	0.5	RS
15	4	0	0.75	RS
16	1	0	0.5	RS
17	1	1	X	RF
18	4	0	0.1	RS
19	4	X	X	X
20	3	X	X	X
21	4	X	X	X
22	1	X	X	X
23	1	X	X	X
24	5	0	0.5	RS
25	4	0	0.5	RS
26	1	0	0.75	RS
27	4	0	0.1	RS
28	4	X	X	X
29	4	X	X	X
30	5	0	0.1	RS
31	4	0	1.5	RS
32	3	0	0.1	RS
33	3	0	0.3	RS
34	4	0	2.9	RS
35	5	1	X	RF
36	3	0	1.9	RS
37	4	0	0.85	RS
38	5	X	X	X
39	3	X	X	X
40	5	0	0.5	RS

RS, relative success; RF, relative failure; MBL, marginal bone loss; X, no dental implant rehabilitation

The mean satisfaction scores for patients rehabilitated by dental implants and other rehabilitation techniques were 3.73 ± 1.19 and 3.21 ± 1.26, respectively. The correlation between the ACS-IABH-Compliance score and final satisfaction was also statistically significant (*p* < 0.05). No significant differences were observed between the satisfaction scores for implanted and non-implanted patients (*p* > 0.05).

## Discussion

Cleft lip and palate treatment may consist of different steps, from the neonatal period to adulthood. Primary surgical repair, nasal repair, bone grafting, orthodontics, and dental rehabilitation procedures are not standardized. The Eurocleft survey, a European inter-center comparison study [29], revealed significant differences in outcomes. Of the 201 centers registered with the network, 194 reported different protocols for treating unilateral clefts. Prosthetic dental rehabilitation is the final step in gaining form and function. To facilitate optimal outcomes, implant therapy in alveolar cleft sites can be considered within a specific timeline and with strategic considerations [30]. This way, an implant survival rate of 95% can be achieved [19, 20, 31, 32]. However, bone level reduction due to anatomic defects or orthodontics problems, periodontium inflammation, bad oral hygiene control, maxillary arch irregularities, and scar tissue folds remain challenging for implant therapy in patients with cleft compared with non-cleft patients [30]. According to Wang et al. [32], implant “success rates” are different from “successful outcomes,” which include function, aesthetics, and inflammation. This study assessed progressive MBL around implants with a post-loading period of a minimum of three years and global patient satisfaction; however, to complete the data, function and esthetic factors should be assessed by clinicians in a future study. In addition, peri-implant soft tissues and prostheses influence the progression of MBL around healthy implants and thus tissue stability. Marginal bone loss is a key consideration and is recognized as a crucial factor in osteointegration. Classically, in non-cleft patients, a 2 mm bone loss around the implant can be assumed to be normal in classification and consensus statements [27, 33]. However, many other studies found maximal bone loss in the first year to be unsuitable, with variability in results: 1.5 mm [34], 1.8 mm [35], 1.8–2 mm [36, 37]. In the current study, in the RS group, an MBL > 1.9 mm was found in cases number 5 and 34 with combined scores of 12 and 15, respectively.

Implants should be preferred for dental cleft rehabilitation [16] because they allow functional loading of the bone graft and provide good esthetic results [38]. Dental prostheses supported by endosseous implants in grafted alveolar clefts have been shown to be a very reliable option in dental rehabilitation for patients with CLP; thus, good long-term results can generally be expected [12]. Furthermore, after treatment with dental implants, satisfaction and oral health quality of life of patients with CLP are comparable to those of the general population [39]. For these patients, bone height assessment (IABH score) alone is frequently used to guide rehabilitation choice, without consideration of soft tissues or adjacent teeth. IABH scoring is a two-dimensional assessment of bone resorption in patient with cleft which is not enough to decide the implant placement. A weak IABH avoid an useless CBCT and irradiation exposure. The implant placement requires a three-dimensional volumetric assessment of the bone graft even if IABH is optimal. In contrast, Deppe et al. [40] showed the importance of the keratinized mucosa for implant success in patients with CLP. Dental prosthesis is obviously not the final step of the treatment for patients with cleft. Dental rehabilitation participates to aesthetic, phonation, mastication, improve the quality of life and therefore psychologic and social qualities. Close multi-disciplinary medical follow-up is necessary to assess it. Our own clinical experience showed that CLP patient cooperation is also critical throughout this multiple operation process. Therefore, we proposed a new combined score “ACS-IABH-Compliance score” that allows an integral preoperative examination of patients with cleft (IABH, alveolar bone height; ACS, width of the prosthetic space, nature of the lateral incisor, state of the adjacent teeth, quality of the keratinized gingiva, quality of the adjacent teeth; Compliance, smoking, hygiene, and observance). In our case series, four patients received four implants (cases number 6, 15, 34, and 25), two cases presented with premaxillary necrosis after primary repair, and two other cases justified tooth extraction close to the cleft area. For these cases, we needed to adapt the IABH score, and the extent of vertical bone height was measured from canine to canine.

This retrospective study aimed to evaluate the impact of preoperative cleft evaluation before dental rehabilitation on relative implant success and patient satisfaction after three years of follow-up. After three years of follow-up, six implants (of 40 placed) were lost. The implant survival rate of 90% is comparable to that reported by Kearns et al. [16] but slightly less than that of other studies, that cited a rate of 95% [19, 20, 31, 32]. Except for case number 5, with a score of 12, that was included in the RS group, all patients with low “ACS-IABH-Compliance” scores (cases number 4, 8, 17, and 35) were in the RF group. Based on these results, we proposed to assess the alveolar cleft status with a combined score and avoid selecting patients with a threshold score < 14 for dental implants. There was a statistically significant difference in average “ACS-IABH-Compliance” scores between the RS and RF groups (*p* < 0.05). This suggests that the ACS-IABH-Compliance cleft score had an impact on relative implant success and highlights the importance of preoperative cleft examination in predicting satisfactory implant outcomes for these patients. High long-term implant success rates, with satisfaction, for patients with CLP were achieved only when prerequisites were completed (high ACS-IABH-Compliance index). This was confirmed in different studies that evaluated different parameters of success and satisfaction [41–43]. A systematic review by Sales et al. [42] showed a high survival rate of dental implants placed in bone graft areas in patients with alveolar clefts. The iliac bone, followed by the mandible, were the most commonly used bones for reconstructive surgery in patients with cleft. Alberga et al. [43] showed that dental implant therapy in patients with cleft is associated with high implant survival, minor MBL, healthy peri-implant soft tissues, and high patient satisfaction; however, the esthetics of the soft tissues were worse in patients with cleft than in augmented non-cleft patients.

Bronstrup et al. [41] evaluated the impact of prosthetic rehabilitation on oral health-related quality of life in patients with congenital or acquired dental and orofacial tissue loss. That mixed-method study compared different criteria for oral quality of life in patients with CLP and prosthetic rehabilitation and patients with an edentulous mandible and/or maxilla treated with implant-supported fixed complete dentures. After rehabilitation, patients with CLP reported improvement in psychosocial functions but worsening in physical functions. Moreover, patients with implant-supported fixed complete dentures saw improvements in all domains; however, paradoxically, they were less satisfied.

To reduce adverse postoperative implant outcomes, medical teams should propose dental implant rehabilitation only for eligible patients with an acceptable ACS-IABH-Compliance score.

Data analysis also indicated that there were no significant differences in final satisfaction scores between the implanted and non-implanted patients. Thus, for low-rated cleft cases without opportunities for improvement, other types of prosthetic rehabilitation can easily be proposed, thereby achieving equivalent final satisfaction ratings. These results are in contrast to those of Papi et al. [44], who revealed that patients rehabilitated with implant-supported dentures and fixed partial dentures showed a better level of satisfaction with their prostheses, while subjects with removable partial dentures showed the lowest satisfaction.

Our outcomes also show that “Sum ACS-IABH-Compliance” is correlated with final patient satisfaction. This suggests that, regardless of prosthetic rehabilitation choice, preoperative conditions must be improved to obtain a high level of satisfaction. The combined score can be increased by improving the bone (bone grafting) and the soft tissue environment, and also provides the best approach for patient motivation toward oral health and hygiene. Nevertheless, patients with cleft endure long surgical protocols from birth and are sometimes victims of dwindling motivation.

A prospective multicenter study may confirm the minimal combined score (14) above which implants could be placed with better postoperative outcomes.

## Conclusion

This retrospective study showed that preoperative parameters, based on the new combined score (ACS-IABH-Compliance), have an impact on implant success and final satisfaction in patients with CLP. This score allows a full preoperative patient evaluation of bone height, soft tissues, and patient cooperation, thereby enabling accurate selection of cleft cases eligible for implants and improving postoperative outcomes. The results of the average “ACS-IABH-Compliance” scores for patients rehabilitated by dental implants was 16.26 ± 2.63 and it was 6.64 ± 2.86 for the other types of rehabilitation and among the patients treated with dental implants the combined score was 16.90 ± 2.35 for the group of patients with success and 12.75 ± 0.43 for the group of patients with failure. Based on these results, we propose the assessment of alveolar cleft status using the combined score and avoidance of dental implants in patients with a threshold score < 14. Where possible, to reduce adverse postoperative outcomes, multidisciplinary medical teams should improve the clinical conditions before dental rehabilitation. Moreover, clinical cases with lower scores should be referred for other prosthetic rehabilitation techniques that can achieve equivalent patient satisfaction.

## Data Availability

The datasets generated and/or analzsed during the current study are available from the corresponding author.

## Abbreviations

CLP: Cleft lip and palate (only lip and palate are affected)
CLA: Cleft lip and alveolar (only lip and alveolar bone are affected)
CLAP: Cleft lip alveolar and palate (lip, alveolar bone, and palate are affected)
ACS: Alveolar cleft score
IABH: Interdental-alveolar bone height
MBL: Marginal bone loss
RS: Relative success
RF: Relative failure
